# Targeting the Stroma in the Management of Pancreatic Cancer

**DOI:** 10.3389/fonc.2021.691185

**Published:** 2021-07-14

**Authors:** Penelope Edwards, Byung Woog Kang, Ian Chau

**Affiliations:** ^1^ Department of Medicine, Royal Marsden Hospital, London, United Kingdom; ^2^ Department of Oncology/Hematology, Kyungpook National University Hospital, School of Medicine, Kyungpook National University, Daegu, South Korea

**Keywords:** pancreatic cancer, stroma, immune checkpoint inhibition (ICI), hyaluronic acid, hedgehog (Hh), bruton kinase inhibitor, SPARC

## Abstract

Pancreatic cancer (PC) presents extremely aggressive tumours and is associated with poor survival. This is attributed to the unique features of the tumour microenvironment (TME), which is known to create a dense stromal formation and poorly immunogenic condition. In particular, the TME of PC, including the stromal cells and extracellular matrix, plays an essential role in the progression and chemoresistance of PC. Consequently, several promising agents that target key components of the stroma have already been developed and are currently in multiple stages of clinical trials. Therefore, the authors review the latest available evidence on novel stroma-targeting approaches, highlighting the potential impact of the stroma as a key component of the TME in PC.

## Introduction

Pancreatic cancer (PC) has a 5-year survival rate of less than 8% (1). The treatment of operable PC involves surgery and adjuvant chemotherapy, yet only around 20% of patients present with an operable disease (2). Even for patients who achieve R0 surgical resection, the likelihood of local relapse is 20% and metastatic disease is 80%. For inoperable patients, chemotherapy is offered, yet the response rate (RR) is generally low with only a marginal improvement in efficacy. In a metastatic setting, triplet therapy includes folinic acid, 5-flurouracil, irinotecan and oxaliplatin (FOLFIRINOX), which has a median overall survival (OS) of 11.1 months (3). Comparatively, the OS for gemcitabine alone is 6.6 months, with a modest improvement to a 8.7 months when adding nanoparticle albumin-bound paclitaxel (nab-paclitaxel) (4, 5). Despite the recent development of new chemotherapeutic regimens, patients with PC still show a dismal prognosis, thus new treatment options are urgently required.

The dense stromal formation surrounding PC and tumour microenvironment (TME) contributes to the aggressiveness of PC (6). The stromal cells consist of cancer-associated fibroblasts (CAFs), immune cells, and endothelial cells, while the extracellular matrix (ECM) includes collagen, fibronectin, glycosaminoglycan, hyaluronic acid (HA), and proteinases (7). In particular, the fibrotic stromal reaction and desmoplastic TME are generally related with the recruitment and activation of CAFs, extensive infiltration of ECM components, and an altered tumour vasculature (8). Current evidence suggests that this stromal remodelling is directly involved in tumourigenesis and cancer progression (9). Moreover, the fibrotic reaction inhibits the immune response, which is impaired as a result of several immunosuppressive mechanisms (10). Therefore, new treatment strategies that target the tumour stroma may be effective for patients with PC.

Cancer research studied the factors that promote normal pancreatic cells to develop into preneoplastic lesions and eventually to invasive cancer (11). Activating mutations have been found in Kirsten Rat Sarcoma Viral Oncogene Homologue (KRAS), p16, and p53, plus around 10% of PC has been found to have a hereditary or genetic component, with germline alterations most commonly related to breast cancer gene 2 (BRCA2) and others (12, 13). Recently, following a comprehensive integrated genomic analysis of 456 pancreatic ductal adenocarcinomas using various platforms, the Cancer Genome Atlas Research Network (TCGA) proposed four distinct subtypes: squamous, aberrantly differentiated endocrine exocrine (ADEX), pancreatic progenitor, and immunogenic tumours (14). Interestingly, the squamous and immunogenic subtypes are especially related to the TME than the tumour cells (11). Another recent investigation also established a stromal classification with two subtypes ECM-rich and immune-rich, where the ECM-rich subtype was associated with a shorter survival compared to immune-rich tumours (15). Similar to this classification, digital deconvolution of transcriptomic data identified two distinct stromal-specific gene expression signatures: normal and activated, where normal stroma was dominated by markers for pancreatic stellate cells (PSCs), whereas activated stroma was characterized by inflammatory signatures associated with a significantly worse prognosis (16). Consequently, based on a better molecular characterization of PC that includes the stroma, targeting the appropriate stromal components may lead to a better outcome and represent a promising approach for precision medicine in PC treatment.

## Role Of Desmoplastic Stroma in Tumour Microenvironment of Pancreatic Cancer

**Figure 1:**
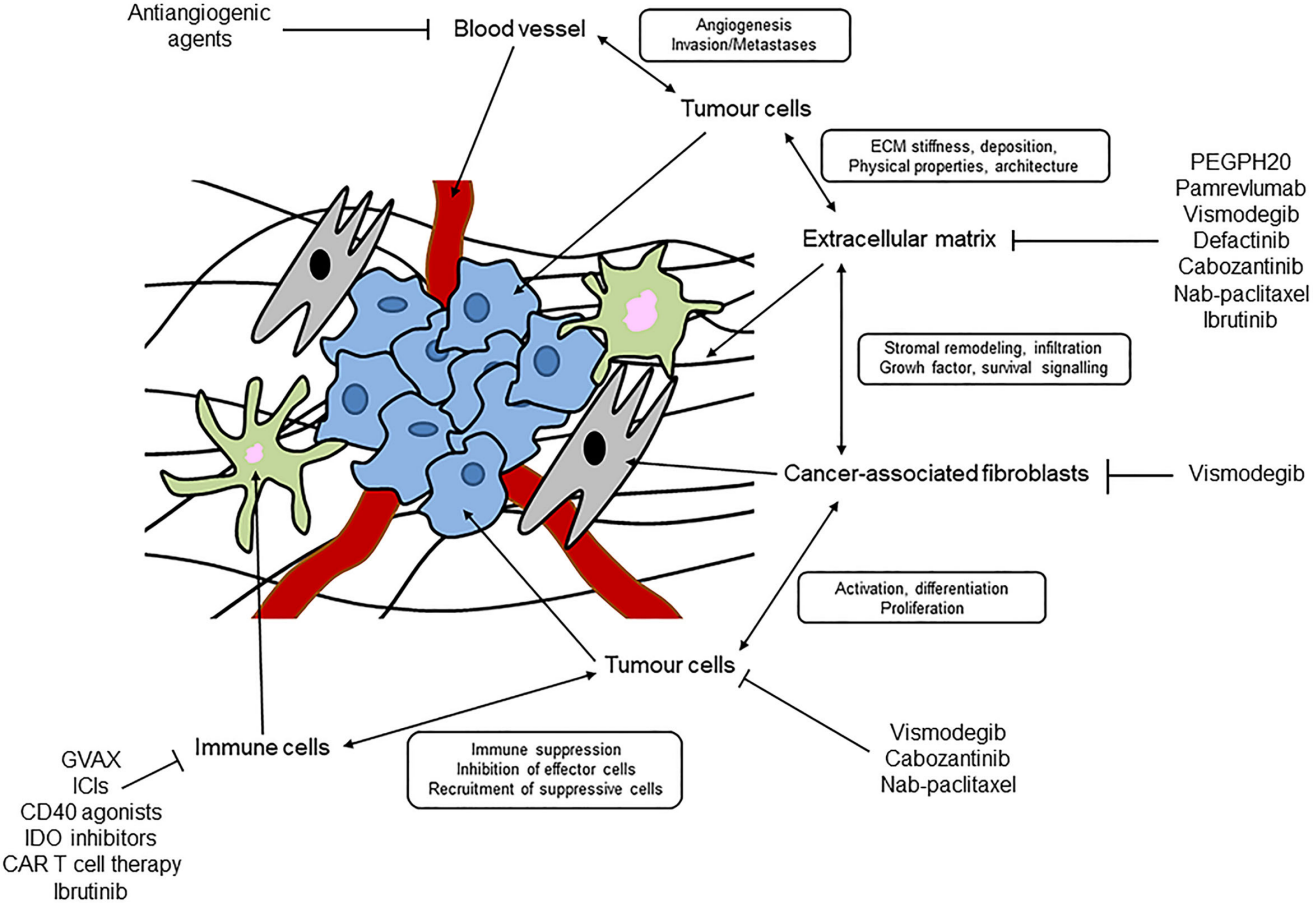
Schematic representation of desmoplastic stroma and tumour microenvironment in pancreatic cancer.

A desmoplastic stroma helps to define PC and is the basis of a complex TME (17). As mentioned above, a desmoplastic stroma is a very complex and heterogeneous network, and organized by interactions among various cell types and acellular components (9) (**Figure 1**).

**Figure 2:**
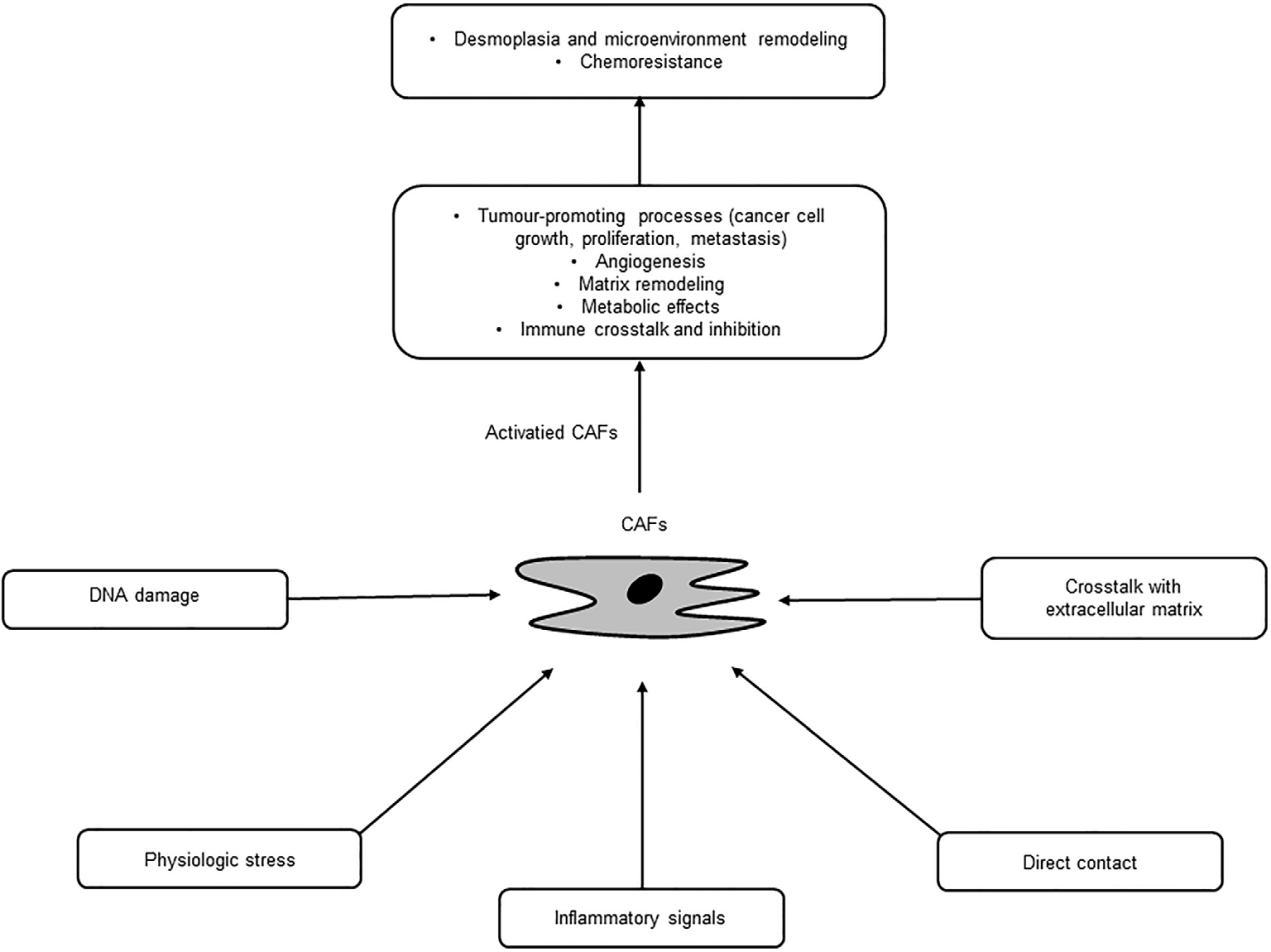
Activation and diverse functions of cancer-associated fibroblasts (CAFs).

CAFs are a major cellular component of PC stroma and derived from several origins, such as PSCs, fibroblasts, and epithelial, endothelial, and mesenchymal cells, through multiple activation pathways including epithelial-mesenchymal transition (EMT) (18, 19) (**Table 1**). PSCs are major sources of CAFs and found in the periacinar, perivascular, or periductal sites of the exocrine pancreas (26–28). In normal conditions, PSCs are quiescent with long cytoplasmic extensions and vitamin-A storing fat droplets (28). In the case of activation, PSCs transform into a myofibroblast-like phenotype that can support pancreatic fibrosis and tumour growth in PC (20). Moreover, adipocytes, pericytes, monocytes, endothelial cells, and bone marrow-derived or adipose-derived mesenchymal stem cells can differentiate into CAFs (21–25). These diverse origin of CAFs may explain their multiple roles and abilities in terms of tumour growth and progression in PC (29). These pathways are inactive in normal tissue, yet activated by secreted factors, such as tumour necrosis factor α (TNFα), transforming growth factor β (TGF-β), platelet derived growth factor (PDGF), interleukins, and sonic hedgehog (SHh), where these activated, specialized forms of fibroblasts express alpha-smooth muscle actin (αSMA), stromal cell-derived factor 1 alpha, fibroblast activation protein, and fibroblast specific protein-1 (7, 30). Once activated, CAFs are known to work as a key component of the TME with diverse functions, including matrix remodelling, metabolic effects, and immune crosstalk (29). CAFs are also a substantial source of growth factors, cytokines and exosomes that can induce the characteristic desmoplasia of the stroma. CAFs eventually promote various tumour-promoting processes, including cancer cell growth, proliferation, metastasis, and tumour angiogenesis and contribute to tumour therapy resistance (31) (**Figure 2**). Ohlund et al. recently identified two distinct populations of inflammatory fibroblasts (iCAFs) and myofibroblasts (myoCAFs) in PC (32). myoCAFs are located in close proximity to tumour cells and express high levels of αSMA, while iCAFs are localized at distal sites and exhibit low levels of αSMA and high levels of inflammatory mediators, contributing to an immunosuppressive environment (18, 29).

**Table 1:** Potential sources of cancer-associated fibroblasts (CAFs).

Origin	Description	References
Pancreatic stellate cells (PSCs)	PSCs are the most studied CAF subtype and may participate in cancer pathogenesis after transforming from a quiescent state into an “activated” state	(20)
Bone marrow-derived mesenchymal stem cells (BM-MSCs)	BM-MSCs can differentiate into a substantial proportion of CAFs in cancers.	(21)
Adipocytes	Human adipose tissue-derived stem cells are a source of CAFs and exhibit the functional properties of CAFs.	(22)
Pericytes	Perivascular cells have been identified as a major source of profibrotic cells in acute liver injury	(23)
Epithelial cells	A substantial number of organ fibroblasts appear *via a* novel direction reversal of the epithelial cell fate.	(24)
Endothelial cells	The endothelial to mesenchymal transition is associated with a phenotype conversion into fibroblast-like cells.	(25)

The ECM contains proteins, such as collagen, integrin, several types of glycans, and multiple soluble factors, with diverse physical and biochemical properties (33). These factors function as a dynamic molecular system that controls cells by altering proliferation, cytoskeletal organization, cellular differentiation, and receptor signalling (34). The predominant glycosaminoglycan is HA, which increases tumour tissue stiffness, promotes metastasis, and regulates tumour immunity (35). Under normal circumstances, the amount of HA is controlled by a balance between synthesis and degradation (36). However, this balance can be shifted towards a higher concentration in PC and this HA‐rich microenvironment can then promote tumour proliferation by enhancing cell growth, migration, invasion, EMT, and metastasis (36, 37). Several studies have reported increased expression of HA and its receptors in PC tissues, which is also associated with poor prognosis (38). *In vitro* studies have also revealed that HA can stimulate PC cell migration. Moreover, animal studies found accelerated tumour growth from the accumulation of extracellular HA due to the forced expression of synthesizing genes (39). In addition to its significant tumour-promoting effects, HA increases the interstitial fluid pressure, thereby compressing intratumoural blood vessels and resulting in hypoxia. This hypoxia may induce angiogenesis and, in an experimental model, the induction of HA overproduction was found to accelerate angiogenesis through stromal cell recruitment (40).

Along with HA, extracellular proteins, including connective tissue growth factor (CTGF) and secreted protein acidic and rich in cysteine (SPARC) are also essential for maintaining physiologic homeostasis and modulate multiple biologic processes, such as wound repair, tissue remodeling, angiogenesis, matrix cell adhesion, cell differentiation, proliferation, and migration (9, 41). The functions of these molecules might be in part mediated by interaction with PSCs/CAFs and several growth factors, like TGF-β and fibroblast growth factor (FGF) (16). Although the role of ECM components in carcinogenesis remains controversial in PC, they are highly expressed in various types of cancer (42). Plus, several studies have demonstrated that overexpression of ECM components generally indicates a worse prognosis in PC (43). Thus, when taken together, ECM components could represent a promising therapeutic target in the treatment of PC.

PC is also characterized by excessive dense ECM deposition associated with vasculature collapse and limited oxygen delivery, promoting the induction of an invasive and treatment-resistant phenotype (44, 45). In response to hypoxia, PC tumour cells produce several angiogenic molecules, including hypoxia-inducible factors (HIFs), vascular endothelial growth factors (VEGFs), FGFs, PDGF, Matrix metallopeptidase 9 (MMP-9), and interleukin-8. Hypoxia-promoted changes lead to increased tumour proliferation, metabolic changes, and also contribute to an immunosuppressive phenotype in PC (46). Multiple studies have already explored the relationship between angiogenic mediators regulated by hypoxia and PC tumour cell survival (47–53). For example, the contribution of HIF-1α and VEGF-A to the aggressiveness of PC is not only *via* angiogenesis but also *via* direct stimulation of tumour cell proliferation and metastatic capacity (45, 54, 55). Furthermore, the abnormal formation of the vasculature inhibits effective drug penetration and uptake, contributing to the lack of efficiency of conventional chemotherapies in PC treatment (56). This explains the attempts to target the hypoxic environment in PC either directly or indirectly

An immune defect in the TME is another interesting aspect for treating PC. Although not fully elucidated, the mechanism of immune suppression may be due to a low immunogenicity, the activation of stromal cells, recruitment of immunosuppressive cells, dense fibrotic stroma, or the secretion of immunosuppressing cytokines (8, 10, 57). PC is known to exhibit a low mutation burden and lack of significant neoantigens, leading to the dysfunction of immune effector cells, such as T cells and natural killer cells (58, 59). CAFs can also reduce activated T cell infiltration of the tumour site and secrete a variety of cytokines, including TGF-β that is associated with EMT (16, 60). This can promote the migration of immunosuppressive inflammatory cells such as regulatory T cells (Treg), myeloid-derived suppressor cells (MDSCs), and tumour-associated macrophages (TAMs) through the CXC motif chemokine ligand (CXCL)-CXC chemokine receptor (CXCR) axis (9, 61). Furthermore, a TME-induced desmoplastic reaction can create a physical barrier for effective T cell migration, thereby evading the immune response. Recent studies have also implicated the role of the intratumoural vasculature which can interact with the immune system and suppress antitumour immunity (8). Therefore, these findings suggest that strategies that can alter the immune suppressive stroma and restore T-cell-mediated immune surveillance may potentially improve the outcome for PC patients.

## Therapeutic Strategies Targeting Stromal Cells in Pancreatic Cancer

Several promising agents that target key components of the stroma have already been developed and are currently in multiple stages of clinical trials in PC (**Table 2**).

**Table 2:** Selected phase II and III studies evaluating stroma-targeting agents in pancreatic cancer.

Target	Agents	Phase	Setting	Treatment arms	N	PE	RR (%)	PFS (months)	OS (months)	Reference
Hyaluronic acid	PEGPH20	II	First-line	PEGPH20/Gemcitabine/Nab- paclitaxel	116	PFS/TE	40	6.0	9.6	(62)
				Gemcitabine/Nab-paclitaxel	113		33	5.3	9.2	
	PEGPH20	III	First-line/HA-high	PEGPH20/Gemcitabine/Nab- paclitaxel	327	OS	34	7.1	11.2	(63)
				Gemcitabine/Nab-paclitaxel	165		27	7,1	11.5	
	PEGPH20	Ib/II	First-line	PEGPH20/mFOLFIRINOX	55	OS	33	4.3	7.7	(64)
				mFOLFIRINOX	56		45	6.2	14.4	
CTGF	Pamrevlumab (FG-3019)	I/II	LAPC	Pamrevlumab/Gemcitabine/Nab-paclitaxel	37	-	30	-	-	(65)
	Pamrevlumab	III	LAPC	Pamrevlumab/Gemcitabine/Nab-paclitaxel	256	Ongoing				NCT03941093
Hh pathway	Vismodegib	II	First-line	Vismodegib/Gemcitabine/Nab-paclitaxel	71	-	40	5.4	9.8	(66)
FAK	Defactinib	I (exp.)	Refractory solid tumour/PC	Defactinib/Gemcitabine/Pembrolizumb	43	Ongoing				NCT02546531
	Defactinib	II	Resectable	Defactinib/Pembrolizumb	36	Ongoing				NCT03727880
HGF pathway	Cabzantinib	I	First- or Second-line	Cabozantinib/Gemcitabine	12	-	-	4.7	10.1	(67)
	Cabozantinib	II	Second-line or more	Cabozantinib/Atezolizumab	29	Ongoing				NCT04820179
SPARC	Nab-paclitaxel Nab-paclitaxel	I/II III	First-line First-line	Gemcitabine/Nab-paclitaxel	67	-	46	7.1	10.3	(68)
				Gemcitabine/Nab-paclitaxel	431	OS	23	6.7	8.5	(5)
				Gemcitabine	430		7	3.7	5.5	
BTK	Ibrutinib	III	First-line	Ibrutinib/Gemcitabine/Nab- palictaxel	211	OS/PFS	29	5.3	9.7	(69)
				Gemcitabine/Nab-palictaxel	213		42	6.0	10.8	
Antiangiogenic agents										
VEGF	Bevacizumab	III	First-line	Bevacizumab/Gemcitabine/Erlotinib	306	OS	13.5	4.6	7.1	(70)
				Gemcitabine/Erlotinib	301		8.6	3.6	6.0	
	Bevacizumab	III	First-line	Bevacizumab/Gemcitabine	302	OS	13	3.8	5.8	(71)
				Gemcitabine	300		10	2.9	5.9	
VEGFR	Axitinib	III	First-line	Axitinib/Gemcitabine	314	OS	12	4.4	8.5	(72)
				Gemcitabine	316		4	4.4	8.3	
VEGFR	Sorafenib	III	First-line	Sorafenib/Gemcitabine	52	PFS	23	3.8	8.0	(73)
				Gemcitabine	52		19	5.7	9.2	
VEGF	Aflibercept	III	First-line	Aflibercept/Gemcitabine	271	OS	-	3.7	6.5	(74)
				Gemcitabine	275		-	3.7	7.8	
Immunotherapy										
Vaccine	GVAX/CRS-207	II	Second-line or more	GVAX/Cy → CRS-207	61	OS	0	-	6.0	(75)
				GVAX/Cy	29		0	-	3.4	
	GVAX/CRS-207	IIb	Second-line or more	GVAX/Cy → CRS-207	29	OS	-	2.4	4.3	(!38)
				CRS-207	31		-	-	4.1	
				Single-agent chemotherapy	30		9.1	2.4	9.1	
ICIs										
CTLA4	Ipilimumab	II	LAPC or metastatic	Ipilimumab	27	-	0	-	4.5	(76)
Dual ICIs										
CTLA4/PD-1	Ipilimumab/Nivolumab	I/II	Solid tumours	Ipilimumab/Nivolumab	-	Ongoing				NCT01928394
CTLA4/PD-L1	Tremelimumab/Durvalumab	II	Second-line	Tremelimumab/Durvalumab	32	RR	3.1	1.5	3.1	(77)
				Durvalumab	33		0	1.5	3.6	
ICIs/CTx										
PD-1/CTx	Pembrolizumab	Ib/II	First-line	Pembrolizumab/Gemcitabine/Nab-paclitaxel	11	CR	27	9.1	15.0	(78)
CTLA4/PD-L1/CTx	Tremelimumab/Durvalumab	II	First-line	Tremelimumab/Durvalumab/Gemcitabine/Nab-paclitaxel	190	Ongoing				NCT02879318
ICIs/Others										
PD-1/Vaccine	Pembrolizumab/GVAX/CRS-207	II	metastatic	Pembrolizumab/CRS-207/Epacadostat ± GVAX/Cy	70	Ongoing				NCT03006302
PD-1/Vaccine	Nivolumab/GVAX/CRS-207	II	metastatic	Nivolumab/CRS-207/GVAX/Cy	93	Ongoing				NCT02243371
CTLA4/PD-1/Vaccine	Ipilimumab/Nivolumab/GVAX/CRS-207	II	metastatic	Ipilimumab/Nivolumab/GVAX/CRS-207	63	Ongoing				NCT03190265
PD-L1/Hyaluronic acid	Atezolizumab/PEGPH20	II	Resectable	Atezolizumab/PEGPH20	40	Ongoing				NCT03979066

N, patient number; PE, Primary endpoint; RR, response rate; PFS, progression-free survival; OS, overall survival; PEGPH20, pegvorhyaluronidase alfa; Nab-paclitaxel, nanoparticle albumin-bound paclitaxel; TE, thromboembolic events; mFOLFIRINOX, leucovorin, 5-fluorouracil, irinotecan, and oxaliplatin; CTGF, connective tissue growth factor; LAPC, locally advanced pancreatic cancer; Hh, hedgehog; FAK, focal adhesion kinase; exp., expansion; PC, pancreatic cancer; HGF, hepatocyte growth factor; SPARC, secreted protein acidic and rich in cysteine; BTK, bruton tyrosine kinase; VEGF, vascular endothelial growth factor; VEGFR, vascular endothelial growth factor receptor; Cy, cyclophosphamide; ICIs, immune checkpoint inhibitors; CTx, chemotherapy; CR, complete response.

### Hyaluronic Acid

Targeting HA deposition can be divided into 3 approaches: depleting stromal HA, inhibiting HA synthesis, and blocking HA signalling (79). In a mouse model, pegvorhyaluronidase alfa (PEGPH20), the PEGylated form of a recombinant human hyaluronidase, has been shown to deplete or reduce HA within the stroma, thereby improving tumour perfusion and drug delivery (80). Moreover, when combined with chemotherapy, PEGPH20 exhibited a synergistic effect with substantially tumour inhibition (81). Such preclinical studies led to a phase Ib study by Hingorani et al. to evaluate the safety and efficacy of escalating doses of intravenous PEGPH20 combined with gemcitabine in patients with metastatic PC (62). The combination was well tolerated and demonstrated therapeutic benefit in 28 patients. Plus, patients with high levels of HA expression showed an improved objective response and median survival. These results then prompted an investigation of PEGPH20 with gemcitabine plus nab-paclitaxel in the phase II HALO 202 trial (82). However, stage one produced an imbalance of thromboembolic (TE) events for patients receiving PEGPH20, therefore, the study protocol was revised to exclude high-TE-risk patients and include prophylactic enoxaparin. In stage two using the revised protocol, the primary endpoints were progression-free survival (PFS) and the incidence of TEs. The randomized study of 279 patients with untreated metastatic PC administered PEGPH20 plus chemotherapy or chemotherapy alone. The patients that received PEGPH20 plus gemcitabine and nab-paclitaxel benefited from an improved median PFS (6.0 *versus* 5.3 months, *P*=0.049) and the TE events were comparable after including enoxaparin. The benefit was even more pronounced in patients with HA-high tumours, showing a median PFS of 9.2 months for the PEGPH20 arm *versus* 5.2 months for the control arm (*P*=0.048). The proportion of patients with HA-high tumours was 34% as defined as extracellular matrix HA staining ≥50% of tumour surface at any intensity. Notwithstanding, the PEGPH20 treatment was also associated with an increased incidence and severity of other manageable adverse events (AEs), such as fatigue, muscle spasms, arthralgia, edema, and neutropenia.

Due to encouraging phase I and II studies, PEGPH20 was investigated in the phase III Halo 301 trial (NCT02715804), which used a combination of PEGPH20 and gemcitabine plus nab-paclitaxel in previously untreated patients with stage IV HA-high PC (63, 83). In this study, the HA status was prospectively determined using a VENTANA HA assay, with HA-high defined as ≥50% staining of a tumour sample. The primary endpoint was OS, with PFS, the objective RR, and safety included as secondary study endpoints. According to predefined criteria, 494 patients with HA-high PC were randomly assigned in a 2:1 ratio to receive first-line chemotherapy with gemcitabine and nab-paclitaxel in combination with either PEGPH20 or a placebo, and prophylactic enoxaparin was given for the TE risk. However, HALO 301 did not meet its primary endpoint of OS (11.2 months *versus* 11.5 months, *P*=0.97) (63, 84). Moreover, the combination arm did not improve the PFS or duration of response. This negative finding could imply that targeting the stromal component can affect resistance to chemotherapies or be attributed to compensatory mechanisms overcoming tumour stroma inhibition, suggesting that targeting desmoplasia alone is not sufficient and a combination of stromal modifying agents is needed. The results from the HALO 301 trial are also consistent with the results from a recently presented randomized phase I/II study (SWOG S1313) evaluating the efficacy of PEGPH20 and modified FOLFIRINOX in patients with metastatic PC. This trial closed early due to an inferior survival and significantly higher AEs in the combination arm (64). Consequently, new studies using PEGPE20 include the addition of immune checkpoint inhibitors (ICIs) (NCT03634332, NCT03979066). The therapeutic potential of PEGPH20 combined with an immune checkpoint inhibitor was studied in a murine synergic breast tumour model (85). The combination of PEGPH20 with oncolytic reovirus therapy and anti-programmed cell death protein ligand-1 (PD-L1)-targeting antibody (anti-PD-L1) resulted in enhanced anti-tumour activity and also extended survival. In addition, PEGPH20 facilitated the infiltration of cytotoxic T lymphocytes and improved the delivery of chemotherapy and programmed cell death protein-1 (PD-1)/PD-L1 antibodies (86). Therefore, these data suggest that the removal of HA by PEGPH20 can increase the efficacy of immune checkpoint therapy and also enhance the accumulation of immunotherapeutic antibodies in HA-rich tumours. Plus, PEGPE20 is also being tested as a main drug in biomarker-driven trials (NCT03193190).

### Connective Tissue Growth Factor

CTGF is a profibrotic extracellular protein with an abundantly elevated expression in PC (16). CTGF is mediated by chemokine signalling, which promotes fibrosis and collagen deposition, which in turn causes cancer progression and metastasis (87, 88). Pamrevlumab (FG-3019), a fully human, recombinant DNA‐derived IgG1 kappa monoclonal antibody against CTGF, attenuates the malignant properties of different human cancers and is currently under clinical trial for the treatment of PC. In a preclinical setting using a mouse model, enhanced antitumour activity was observed when using pamrevlumab as a single agent or in combined with gemcitabine (50, 89). In a phase I study, the safety and efficacy of increasing doses of pamrevlumab were evaluated in combination with two chemotherapy agents, gemcitabine and erlotinib, in 75 patients with previously untreated Stage III/IV PC (65). The results showed that pamrevlumab was well tolerated with no dose-limiting toxicity or dose-related trends in the type or incidence of serious AEs. In a subsequent phase I/II study using gemcitabine/nab-paclitaxel with pamrevlumab or gemcitabine/nab-paclitaxel alone for patients with locally advanced PC, pamrevlumab combination arm showed a higher percentage of surgical resection (33.3% *versus* 7.7%) and improved median survival rate, plus the combination was feasible and well tolerated with no incremental safety signals (90). Accordingly, a phase III (LAPIS) trial is currently ongoing to investigate the efficacy of pamrevlumab plus gemcitabine with nab-paclitaxel in locally advanced, unresectable PC (NCT03941093).

### Hedgehog Pathway

The Hh signalling pathway is one of twelve core signalling pathways and processes in PC and plays a critical role in regulating the dense stroma of pancreatic cancer *in vivo* and *in vitro* (91, 92). Saridegib (IPI-926) is a potent and specific inhibitor of smoothened (Smo), a key signalling transmembrane protein in the Hh pathway (93). Preclinical studies using a mouse model showed that saridegib depleted tumour-associated stromal tissue and increased the intratumoural mean vessel density, leading to a decreased tumour burden and prolonged survival (94). However, subsequent human clinical trials failed to show a sustained benefit for this agent (95, 96). Vismodegib (GDC-0449) is another Hh pathway inhibitor that blocks Hh signalling by binding to Smo (93). Similarly, in contrast to preclinical results, the addition of vismodegib to chemotherapy did not improve the overall outcomes in phase I and II studies (66, 97). Therefore, these disappointing results suggest that inhibiting the Hh pathway ironically stimulates aggressive PC clones and invigorates the metastatic capacity (59). Plus, the inhibition of CAFs *via* suppressing the Hh signalling pathway may induce a decreased immune response and vascular dysfunction, revealing the complex relationship between Hh signalling and desmoplastic stroma (91). Therefore, there are currently no ongoing studies on Hh signalling inhibitors in PC, except for the biomarker-driven trial (NCT02465060).

### Focal Adhesion Kinase

Focal adhesion kinase (FAK) is a nonreceptor protein tyrosine kinase that plays a central role in the collagen pathway and regulates integrin-mediated ECM signalling (98). FAK is frequently overexpressed and dysregulated in a variety of cancers, including PC, and correlated with poor prognosis (98, 99). Interestingly, in preclinical studies, FAK activation induced a fibrotic and immunosuppressive TME, while inhibition of the FAK pathway was found to interrupt the aggressive effects of collagens on PC biology (100, 101). Therefore, these observations led to an investigation of a combination therapy of FAK inhibitors with gemcitabine and pembrolizumab, a humanized monoclonal antibody directed against PD-1. Defactinib (VS-6063) is a selective, orally active, competitive adenosine triphosphate (ATP) inhibitor of FAK. A recent phase I/IIa clinical trial evaluated a dose escalation of defactinib in combination with pembrolizumab and gemcitabine for patients with advanced solid tumours, and subsequently at an optimal dose for an expanded patient cohort with PC (102). Preliminary data have showed that the combination therapy was well-tolerated with no dose-limiting toxicity (DLT), and the common treatment-related AEs included fatigue, nausea, myalgia, vomiting, anorexia, pruritus, and fever. The expanded patient cohort is currently being investigating with pending correlative and efficacy data (103). Another phase II study of defactinib plus pembrolizumab is also ongoing for resectable PC patients (NCT03727880).

### Hepatocyte Growth Factor Pathway

Hepatocyte growth factor (HGF), also known as scatter factor, is the ligand of c-Met (mesenchymal-epithelial transition tyrosine kinase receptor or HGF receptor) that is often secreted by stromal cells and regulates the stromal-tumour interactions in PC (104). The binding of HGF to c-Met leads to the dimerization and phosphorylation of c-Met and triggers several downstream signallings (105). This axis also plays an important role in the pathogenesis of pancreatic cancer and its progression. C-Met overexpression has already been identified in PC and correlated with aggressive disease (106), while elevated serum HGF levels have been associated with disease progression (107). Plus, c-Met activation has been linked with resistance to gemcitabine (108). Interestingly, *in vitro* and *in vivo* models have shown that crizotinib, an ATP competitive multi-target protein kinase inhibitor, increases sensitivity to gemcitabine and inhibits peritoneal dissemination (109, 110). A recent preclinical study demonstrated that the inhibition of both HGF and c-Met combined with gemcitabine improved the reduction of tumour volume and metastasis (105). Another report also indicated that long-term treatments involving cabozantinib induced less resistance and even improved the efficacy of gemcitabine (108). Therefore, these preclinical studies led to a phase I trial of cabozantinib, an orally bioavailable c-Met inhibitor, and gemcitabine in advanced PC (67). This trial involved the treatment of 12 patients and no maximum tolerated dose was determined, plus the DLT was relatively high at all dose levels. Another phase II clinical trial investigated the safety and antitumor activity of cabozantinib combined with atezolizumab in patients with PC (NCT04820179). Thus, several preclinical and early clinical studies have tested the inclusion of c-Met inhibitors, such as rilotumumab, crizotinib, capmatinib, and tivantinib, in combination treatments for solid tumours (104). However, the remaining major challenge is to identify potential predictive biomarkers to facilitate the appropriate selection of specific patient populations most likely to benefit from combination therapies.

### Secreted Protein Acidic and Rich in Cysteine

SPARC is a multifunctional calcium-binding glycoprotein which modulates interactions between cells and the extracellular environment, thereby participating in cell development, matrix cell adhesion, wound repair, tissue remodeling, and angiogenesis (41, 111). SPARC may also influence PC cell proliferation, migration, metastatic, and escape mechanisms (112). Thus, SPARC has been investigated as a potential therapeutic target, where the presence of SPARC in the tumour stroma could be used to deliver albumin-conjugated molecules into the tumour and TME. Meanwhile, nab-paclitaxel, a nanoparticle form of paclitaxel, is known to deplete tumour stroma through interaction between albumin and SPARC (5). Plus, nab-paclitaxel has exhibited clinical antitumour activity in several cancer types that overexpressed SPARC (43, 113). In metastatic PC, nab-paclitaxel was used in multiple-stage clinical trials, where the addition of nab-paclitaxel to gemcitabine showed activity and efficacy in first-line treatment, improving survival and the overall RR (112, 114). Interestingly, patients with high stromal-SPARC expression exhibited a significant increase in OS compared to patients with low stromal-SPARC expression (17.8 *versus* 8.1 months), representing a significant predictor of OS in a multivariate analysis (68). Based on these results, a large, open-label, international, randomized, phase III trial (Metastatic Pancreatic Adenocarcinoma Clinical Trial, MPACT) followed with 861 metastatic PC patients as a first-line setting (5). The patients were randomly assigned 1:1 to receive either nab-paclitaxel plus gemcitabine or gemcitabine alone. As mentioned above, since the trial met its primary endpoint of OS, this combination is now a standard regimen in the treatment of metastatic PC. However, the stromal and tumour levels of SPARC, measured by immunohistochemistry, showed no association with survival (115, 116). These conflicting results on SPARC as a prognostic and predictive biomarker in PC may have been impacted by different methodologies of protein detection and staining evaluation, the origin of the tumour samples, and the level of the cutoff value. Moreover, recent data has suggested that specific tumour delivery of nab-paclitaxel is not directly related to SPARC expression, and nab-paclitaxel does not usually deplete tumour stroma (117). Furthermore, the efficacy of nab-paclitaxel may be dependent on the drug internalization by TAMs (118). Consequently, for PC patients, the potential link between SPARC expression and treatment efficacy remains unclear, and more precise methods are needed to analyse SPARC expression. Plus, discovering a novel targeted nanoparticle and enhanced drug delivery system using SPARC could improve the pharmacologic and therapeutic properties of conventional cancer treatment for patients with PC.

### Bruton Tyrosine Kinase

Ibrutinib is a first-generation Bruton tyrosine kinase (BTK) inhibitor that irreversibly binds to cysteine at position 481 in the kinase domain and blocks enzyme activity (119). Protein tyrosine kinase BTK, which is essential for B cell maturation and expressed by other immune cell populations, has been implicated in the immune regulation and function of myeloid cells (120, 121). BTK has also been shown to exhibit anti-fibrotic effects in PC by effectively inhibiting the infiltration of mast cells in both transgenic mice and patient-derived xenograft models. Furthermore, ibrutinib reduces stromal fibrosis and inhibits tumour progression (122). Preclinical data for ibrutinib plus gemcitabine showed enhanced antitumour activities *versus* gemcitabine alone in both transgenic mouse and patient-derived xenograft PC models (122). Early developmental clinical trials also showed preliminary efficacy in solid tumours, including PC (NCT02562898) (123). Thus, based on these encouraging results, ibrutinib was included in a recent randomized phase III RESOLVE trial (NCT02436668) that assessed ibrutinib in combination with gemcitabine and nab-paclitaxel or a placebo plus chemotherapy as the first-line treatment for patients with metastatic PC (124). Although the full results have yet to be published, it has been revealed that the study failed to meet its primary endpoint of prolonged PFS and no statistically significant PFS or OS benefit was shown when adding ibrutinib to chemotherapy (69). Although both treatment arms showed a similar proportion of patients with grade ≥3 AEs, the patients in the ibrutinib arm were treated for a shorter time period and received lower cumulative doses of all agents compared with the placebo arm. Therefore, this emphasizes the need for clinical biomarkers to predict the sensitivity and resistance to BTK inhibitors and identify the best combination partners for synergistic effects and lower toxicity. Despite these disappointing results, more recent studies are investigating the combination of BTK inhibitors with ICIs. Indeed, multiple cell types, such as MDSCs, macrophages, dendritic cells, and endothelial cells, in the TME are regulated by BTK (119). In a preclinical study, ibrutinib treatment successfully reprogrammed macrophages for increasing CD8+ T cells to assist with tumour control in PC (125). Moreover, the synergistic activity of ibrutinib with ICIs has also been described in preclinical models, including regulating tumour-induced immune tolerance by enhancing the activity of tumour-infiltrating cells and reducing the secretion of immunosuppressive cytokines (126). A recent phase Ib/II study evaluated ibrutinib plus durvalumab, a PD-L1-targeting antibody, in patients with relapsed or refractory solid tumours (127). Although ibrutinib plus durvalumab had an acceptable safety profile, the combination showed limited activity with an overall RR of 2% and median OS of 4.2 months for 49 PC patients. Another randomized phase II study evaluated the effect of a second-generation BTK inhibitor in patients with advanced PC using acalabrutinib alone or in conjunction with pembrolizumab, an anti-PD-1 antibody (128). The monotherapy and combination treatments both showed minimal clinical benefit. Therefore, additional trials are needed to refine the dual inhibition of BTK and the PD-1/PD-L1 pathway in PC.

### Angiogenesis

Various stromal components, such as collagen, fibronectin, HA, VEGF, TGF-β, and CTGF are closely associated with a fibrotic and hypoxic tumour condition, which forms a hard mass and releases several proangiogenic factors, including VEGF, MMP-9, interleukin-8, and fibroblast growth factor-2 (129). These stromal cells and ECM play a crucial role in stimulating or inhibiting angiogenesis *via* multiple pathways and numerous genetic alterations in PC (56). Several studies have already demonstrated that overexpression of VEGF is related with tumour progression and a poor prognosis, and antiangiogenic treatment reduces tumour cell growth (130). Consequently, anticipating that antiangiogenic therapy could be effective for PC, a single-arm phase II trial with a combination of bevacizumab and gemcitabine did show clinical benefits (131). However, in two large phase III studies, the addition of bevacizumab to chemotherapy failed to reach the primary endpoint of OS (70, 71). Despite several subsequent clinical trials using bevacizumab and other chemotherapy backbones, no treatment benefits were reported (130).

Moreover, subsequent phase III clinical trials of antiangiogenic agents targeting vascular endothelial growth factor receptor (VEGFR) or VEGF, including axitinib, sorafenib, and aflibercept, showed no significant prolongation of OS (56, 72–74). Such findings are also consistent with two meta-analyses, that indicated no increase in OS with any VEGF or VEGFR-targeting treatment (130, 132). There are various explanations for the limited clinical outcomes of antiangiogenic agents in PC (133). Antiangiogenic therapy may inhibit the blood supply to tumours and inevitably diminish the drug delivery, which would partially explain the combined need for inhibition of angiogenesis and alternative mechanisms in the treatment of PC (134). Antiangiogenic therapy per se may support a conversion to a more aggressive phenotype, promoting the induction of invasive and treatment-resistant tumours (129). In addition, the angiogenesis pathway comprises a complex network of crosstalk with many parallel cascades, so its inhibition may induce compensatory upregulation of proangiogenic factors, thereby paradoxically increasing tumour growth (56). Moreover, an adaptive response to hypoxia primarily mediated by HIFs confers more aggressive phenotypes in PC cells (129). Finally, continuous hypoxic microenvironment remodelling can stimulate the regulation of autophagy and generation of reactive oxygen species and alternative pathways, including the metabolism of glucose or glutamine, while also contributing to therapeutically resistant behaviour of PC (135). Therefore, considering its complex and multifaceted functionality, further studies of antiangiogenic therapy in PC are needed to identify the best combination partners for synergistic effects, especially with stromal depletion strategies that target specific stromal features. Exploring potential biomarkers will also be important to select the appropriate PC patient populations.

### Immune Cells

The ability to evade immune surveillance is a recognized hall mark in PC biology (136). Since PC is also well characterized by immune-quiescent desmoplastic stroma and the dominance of immunosuppressive cells, some recent research has been focusing on the development of immune-based therapies for PC (57). One promising immunotherapeutic strategy is to stimulate T-cell priming and dendritic cell activation, as the resulting activation of tumour-specific T cells and their migration into the TME may be the key to surmounting innate immune suppression and correcting the lack of effector T cells (17, 58). One of the most widely evaluated PC vaccines is GVAX, which is an irradiated allogeneic whole tumour cell vaccine engineered to express a granulocyte-macrophage colony-stimulating factor (137). In early phase studies, GVAX has been found to be tolerable and effective in promoting the development of antitumour immunity (138, 139). These results then prompted a randomized phase 2 trial evaluating GVAX pancreas in combination with cyclophosphamide followed by CRS-207, a live-attenuated listeria vaccine expressing a PC-associated antigen mesothelin in previously treated metastatic PC. This triple combination therapy demonstrated a longer OS with minimal toxicity and enhanced mesothelin-specific CD8 cytotoxic cells correlated with the improved OS (75). However, a subsequent phase IIb trial (ECLIPSE) failed to show any superiority of this combination over chemotherapy (140). This may have been due to the failure of sufficient T cell induction or quick exhaustion of effective immune cells (58). Plus, major contributors, including CAFs, MDSCs, or TAMs may regulate multiple immunosuppressive mechanisms, indicating the dynamic nature and heterogeneity of the immune response in PC (18, 57). Thus, the addition of a new combination approach seems to be the ideal strategy to achieve a more impressive response in PC, and ICIs could be such a treatment.

ICIs can lead to T cell activation, thereby generating an effective immune response. Nevertheless, a broad spectrum of clinical trials for each ICI monotherapy has shown limited clinical success. For example, a phase II study with ipilimumab, a monoclonal antibody targeting cytotoxic T-lymphocyte-associated protein 4 (CTLA-4), showed no significant improvement in survival (76). In early clinical trials, other single-agent anti-PD-1 agents, including pembrolizumab and nivolumab have also been ineffective in the treatment of PC (141, 142). Consequently, combination approaches have been explored to enhance the immune responses for better therapeutic effects when treating PC. For a dual inhibition setting, a recent randomized phase II study evaluated 65 previously treated metastatic PC patients for the efficacy of durvalumab alone or in combination with the anti-CTLA-4 antibody, tremelimumab (77). Although the combination therapy produced a modest improvement with a 3-month disease control rate (DCR) of 9.4%, as the desired threshold efficacy (10%) was not achieved, the study was closed. Another ongoing clinical study is evaluating ipilimumab in combination with nivolumab (NCT01928394) (137).

ICIs in combination with chemotherapeutic agents are an increasing focus as an alternative option for the treatment of PC. A recent phase Ib trial reported that an ipilimumab and gemcitabine combination was safe, including a delayed response in one patient (143). Tremelimumab plus gemcitabine has also demonstrated a tolerable toxicity with median survival of 7.4 months (144). Moreover, the preliminary results from a randomized phase 2 trial (CCTG PA.7) evaluating the efficacy of gemcitabine and nab-paclitaxel in combination with durvalumab and tremelimumab showed a median PFS of 2.5 months and median OS of 8.5 months (NCT02879318) (145). Plus, pembrolizumb was combined with gemcitabine and nab-paclitaxel in a phase Ib/II study including 17 patients with metastatic PC. Of the 11 evaluable chemotherapy naïve patients, this combination showed an interesting outcome with a DCR of 100% and median PFS of 9.1 months, which seems to be much better than typically reported for first-line chemotherapy (78).

Combining of ICIs with a vaccine or other immunomodulatory molecules is another potentially effective regimen, and is currently under clinical investigation to determine the efficacy in PC. Le et al. evaluated combining ipilimumab and the GVAX vaccine in a phase Ib trial, where 30 patients were randomly assigned to single-agent ipilimumab *versus* ipilimumab combined with GVAX. As a result, the combination patients showed a better 1-year OS benefit *versus* ipilimumab alone (27% *versus* 7%), prompting additional GAVX clinical trials with various modalities (146). Numerous similar clinical trials are also underway, including agonist CD40 antibodies, indoleamine 2,3-dioxygenase inhibitors, BTK inhibitors, anti-lymphocyte activating gene-3 monoclonal antibodies, CXCL/CXCR inhibitors, FAK inhibitors, T cell immunoglobulin-related agents, and adoptive cell transfer therapy (so-called ‘CAR T-cell therapy’). Although most of these trials are still ongoing, combined strategies using different therapeutic approaches with ICIs may provide supporting evidence to optimize PC treatment using ICIs (137, 147).

## Discussion and Perspective

Despite substantial advances in the development of new agents and an improved understanding of PC genetics, cytotoxic-backboned chemotherapy remains the key treatment with only a few novel agents being translated into clinical practice. Current research is focused on targeting the specific component of the PC tumour stroma, yet the results so far are disappointing, and there have been few significant advanced in antiangiogenic therapy or immunotherapy. The PC microenvironment is characterized by increased desmoplasia and energy metabolic disorders (28). Plus, accumulating evidence has revealed that the PC stroma is enriched with CAFs or PSCs that produce excessive amounts stromal elements and a matrix, leading to a desmoplastic process (59). These components are also responsible for the generation of a rigid barrier that results in elevated tumour pressure, a hypovascular microenvironment, and attenuated drug delivery (17). A rich stroma and severe fibrotic reaction have also been shown to play a role in remodelling the TME (28). In addition to the mechanical factors, TME alterations in PC can also support the metabolism of cancer cells, even in a nutrient-depleted and hypoxic TME. In particular, an amplified desmoplastic reaction can significantly impair the immune response and augment the immune tolerance (8). Thus, recent data also emphasizes the importance of multiple targeted approaches, including stromal depletion and immune modulation. Therefore, proactively designed combination strategies are needed for more successful development of stroma-targeting agents. Plus, an improved understanding of the interaction complexity between tumour cells and the heterogeneous TME components will help to identify stromal-based biomarkers for categorizing patients who can receive clinical benefit in PC.

Molecular classification could also help to improve patient selection and enable better differentiation of responders and non-responders. For example, as an immunogenic subtype exhibits an upregulation of immune-related genes, this subtype could feasibly be treated with ICIs (148). Meanwhile, a basal-like subtype may benefit more from gemcitabine-based regimens than from FOLFIRINOX (149). Plus, the presence of pancreatic stellate cells in an ADEX subtype could also imply resistance to gemcitabine or radiotherapy (150). More recently, Tiriac et al. performed drug sensitivity profiling of PC organoid models and identified novel functional subtypes for defining gene expression signatures to predict chemotherapy sensitivity (151). Although further studies are required to validate these results in PC, designing and testing novel specific targeted therapies for each subtype may help to avoid unnecessary treatments and expedite the application of an effective drug combination.

## Author Contributions

PE and BK drafted the manuscript. IC managed and reviewed the manuscript. All authors contributed to the article and approved the submitted version.

## Funding

PE and IC would like to acknowledge National Health Service funding to the National Institute for Health Research Biomedical Research Centre at the Royal Marsden NHS Foundation Trust and The Institute of Cancer Research.

## Conflict of Interest

IC: Advisory Board: Eli-Lilly, Bristol Meyers Squibb, MSD, Bayer, Roche, Merck-Serono, Five Prime Therapeutics, Astra-Zeneca, Oncologie International, Pierre Fabre, Boehringer Ingelheim. Research funding: Eli-Lilly, Janssen-Cilag, Sanofi Oncology, Merck-Serono. Honorarium: Eli-Lilly.

The remaining authors declare that the research was conducted in the absence of any commercial or financial relationships that could be construed as a potential conflict of interest.
